# Author Correction: Inhibition of autophagic flux differently modulates cannabidiol-induced death in 2D and 3D glioblastoma cell cultures

**DOI:** 10.1038/s41598-021-98292-2

**Published:** 2021-09-14

**Authors:** Vladimir N. Ivanov, Peter W. Grabham, Cheng-Chia Wu, Tom K. Hei

**Affiliations:** Center for Radiological Research, Department of Radiation Oncology, Vagelos College of Physicians and Surgeons, Columbia University, New York, NY 10032 USA

Correction to: *Scientific Reports*10.1038/s41598-020-59468-4, published online 14 February 2020

The original version of this Article contained errors in Figures 1 and 10, and in the Supplementary Information file. Figure 1a was assembled with two incorrect images of western blots for “ATM” and “p53-P(S15)”. Figure 1b was assembled with an incorrect image of western blot “LC3” at 24 h (left side), which contained an error in treatment condition and a transfer error for lane 1. The corresponding data for Figures 1 and 10 were also corrected in the Supplementary Information file. In addition, an error in treatment conditions for the western blots in Figure 10 was discovered. As a result, the western blots for “TS543”, “CBD (40)” in panel c were removed.

**Figure 1:**
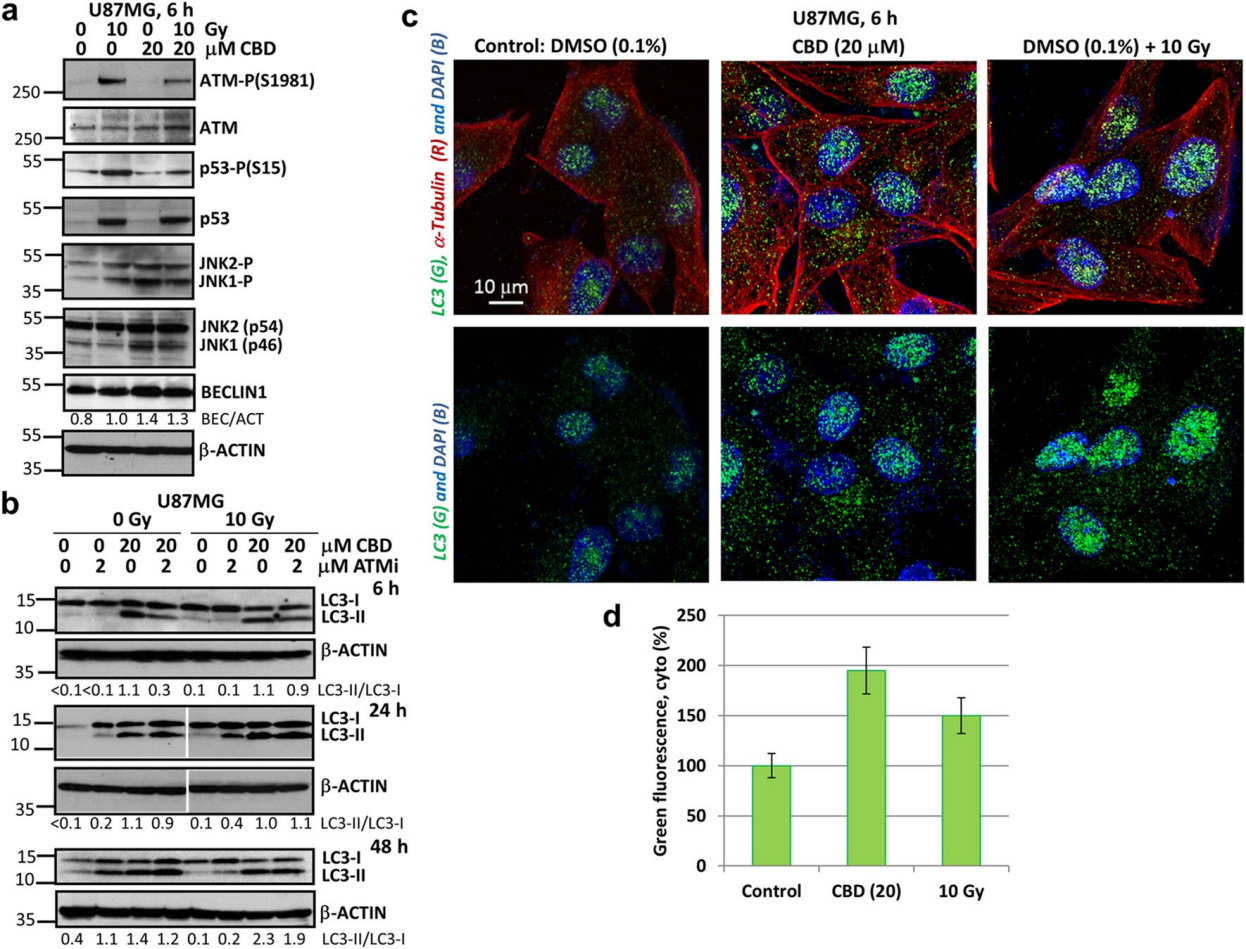
CBD induced autophagy in U87MG GBM 2D cell culture. (**a**) Western blot analysis of cell signaling proteins was performed 6 h after specified treatment of U87MG cells with CBD (20 μM) and γ-irradiation (10 Gy), alone or in combination. (**b**) Western blot analysis of LC3-II and LC3-I autophagy-related proteins was performed 6 h, 24 h and 48 h after treatments of GBM cells with CBD (20 μM), ATMi (2 μM) and γ-irradiation (10 Gy), alone or in combination. Original blots are shown in the “Supplementary information” section. After protein transfer, blot membranes were cut in two (or three) parts, which contained high molecular weight and low molecular weight proteins, respectively. The delineation of membranes was based on the well-known apparent molecular weight of investigated proteins. Cutting membranes were utilized for incubation with corresponding primary antibodies. The center lanes in LC3-I/II and β-ACTIN 24 h blots (which contain protein sample after an additional treatment non-used in this paper) were removed. (**c**) The LC3 puncta formation after indicated treatments of U87MG cells was detected using confocal microscopy with anti-LC3 Ab (green), anti-α-Tubulin Ab (red) and DAPI (blue). Images are shown with scale bar = 10 μm. (**d**) Relative levels of cytoplasmic green fluorescence (LC3) were determined using confocal images.

**Figure 10:**
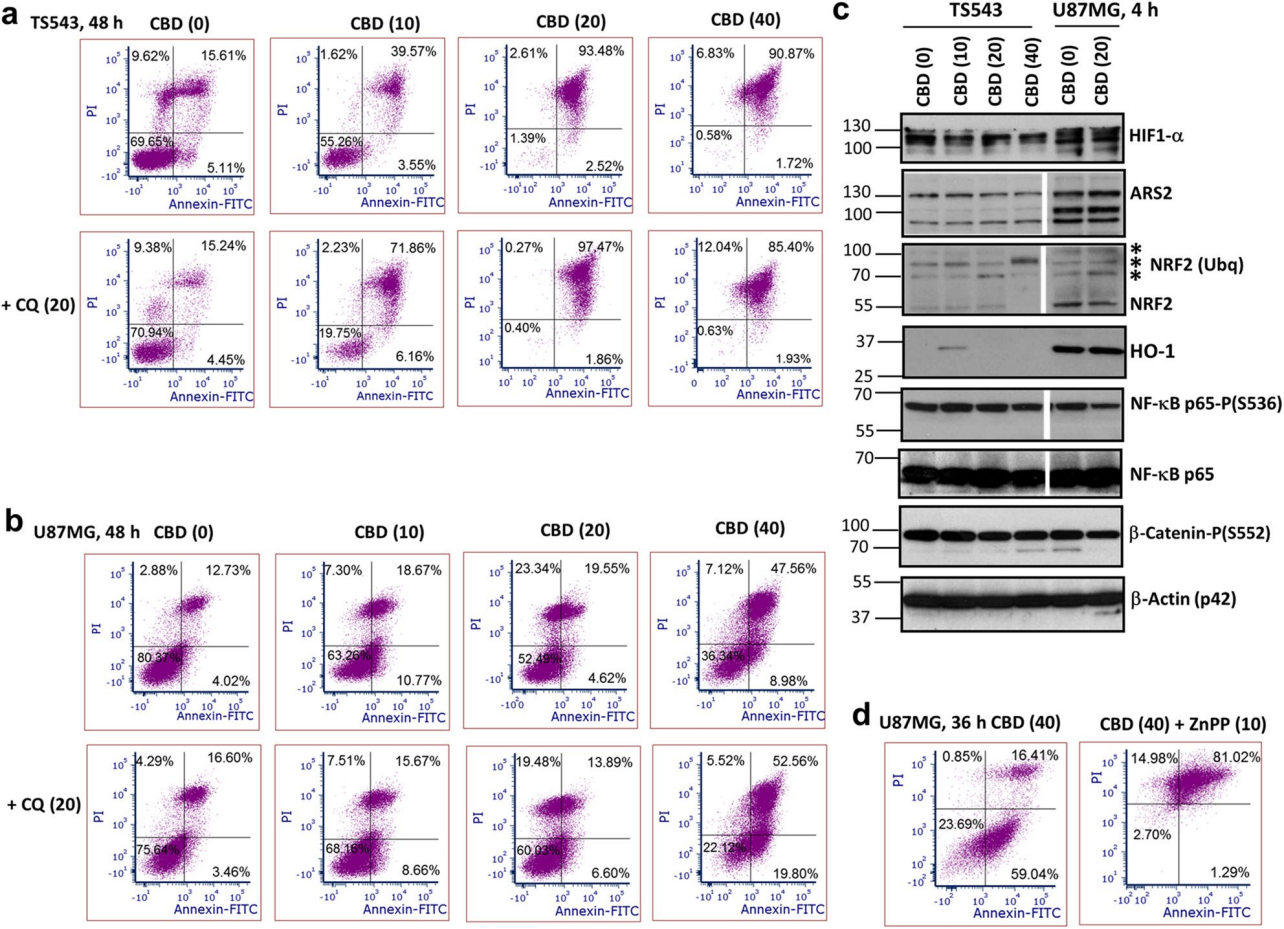
Distinct effects of CBD treatment on TS543 spheroids and U87MG adherent culture. (**a**,**b**,**d**) Annexin-V-FITC and PI staining for determination of the early apoptotic, late apoptotic and secondary-necrotic cells after treatment by CBD (0–40 μM) or CBD+CQ (20 μM) was followed by the flow cytometry. ZnPP (20 μM), an inhibitor of heme oxiginase-1 (HO-1) activity, was added 30 min before CBD. (**c**) Western blot analysis of indicated proteins from 3D TS543 and 2D U87MG cell cultures 4 h after indicated treatments. Original blots are shown in the “Supplementary information” section. After protein transfer, blot membranes were cut in two (or three) parts, which contained high molecular weight and low molecular weight proteins, respectively, and utilized for incubation with corresponding primary antibodies. Two center lanes in gel blots for ARS2, NRF2, NF-κB p65-P and NF-κB p65 (which contain proteins after additional treatments non-used in this paper) were removed. Stars indicate ubiquitinated NRF2.

Consequently, in the legend of Figure 10,

“Two center lanes in gel blots for ARS2, NRF2, NF-κB p65-P and NF-κB p65 (which contain proteins after additional treatments non-used in this paper) were removed. Stars indicate ubiquitinated NRF2.”

now reads:

“The center lanes in western blots (which contain proteins after additional treatments non-used in this paper) were removed.”

The original Figures 1 and 10 and accompanying legends and the original Supplementary Information file are provided below. The original Article and accompanying Supplementary Information file have been corrected.

### Supplementary Information

Supplementary Information.

