# Supplementary material for: Author Correction: Inhibition of autophagic flux differently modulates cannabidiol-induced death in 2D and 3D glioblastoma cell cultures
Source: Sci Rep. 2021 Sep 14;11:18616. doi: 10.1038/s41598-021-98292-2 (PMC8440618; doi:10.1038/s41598-021-98292-2)
Supplement: Supplementary file 1 — Supplementary Information. [file 41598_2021_98292_MOESM1_ESM.pdf]

Inhibition of autophagic flux differently modulates cannabidiol-induced death in 2D and 3D glioblastoma cell cultures

Vladimir N. Ivanov, Peter W. Grabham, Cheng-Chia Wu and Tom K. Hei

Center for Radiological Research, Department of Radiation Oncology,
Vagelos College of Physicians and Surgeons, Columbia University, New York, NY 10032

Correspondence to: *Vladimir N. Ivanov, E-mail address: vni3@cumc.columbia.edu*

Keywords: glioblastoma; cannabidiol; radiotherapy; autophagy, 3D glioma culture

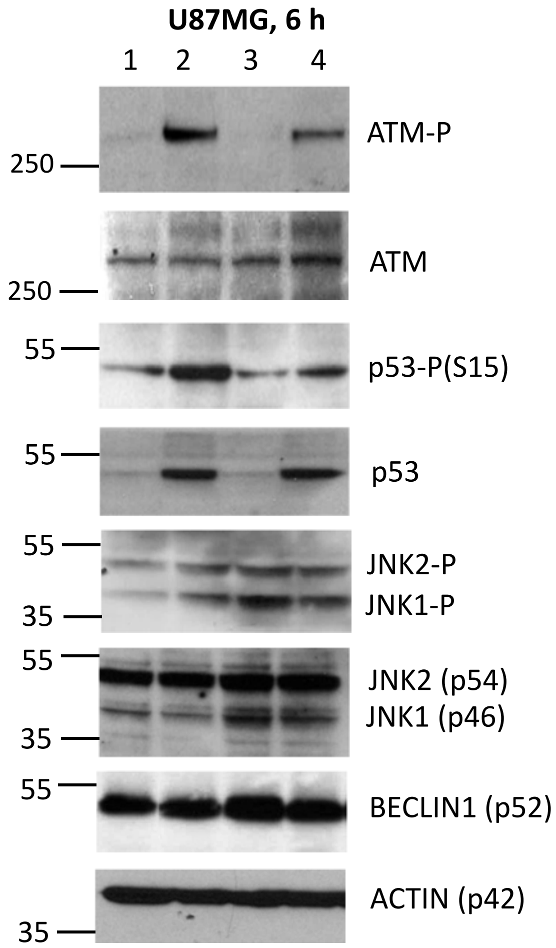

Figure 1a

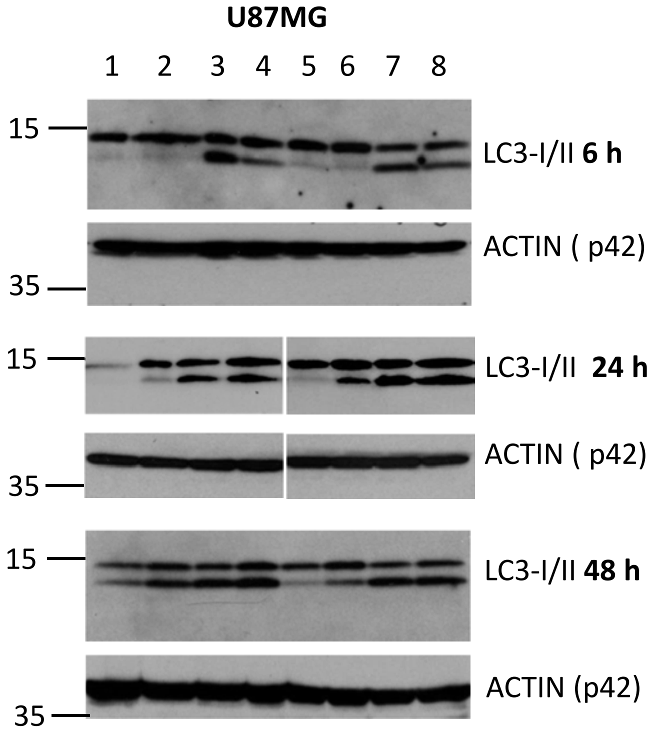

Figure 1b

Figure S1. The originals of Western blots for Figure 1. The center lane in LC3-I/II and ACTIN 24 h blots (which contain protein sample after an additional treatment non-used in this paper) was removed.

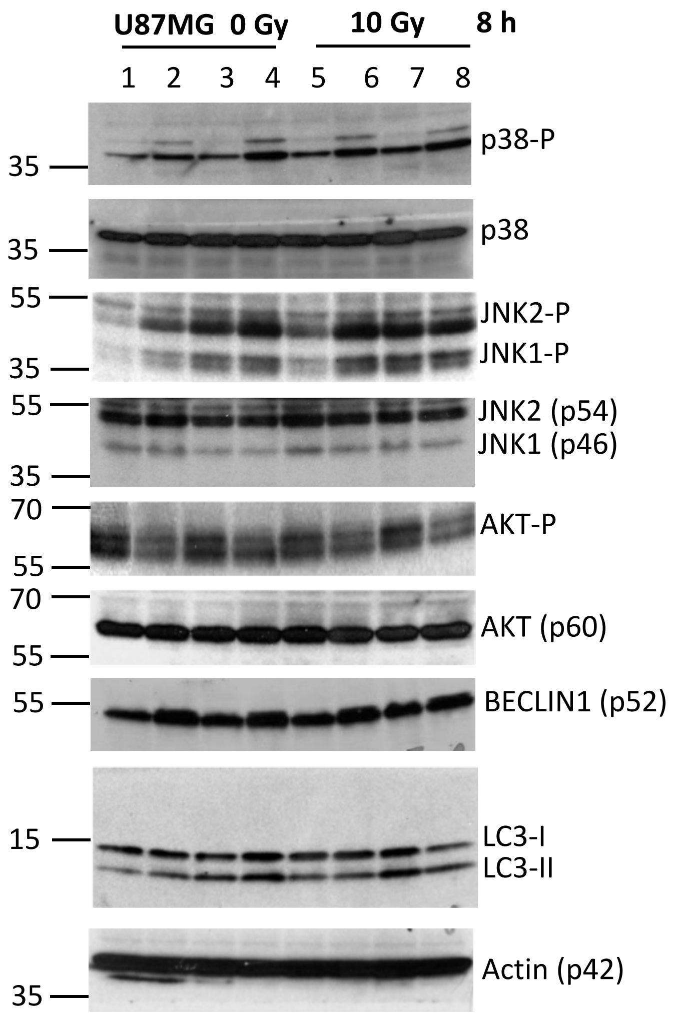

Figure S2. The originals of Western blots for Figure 2a.

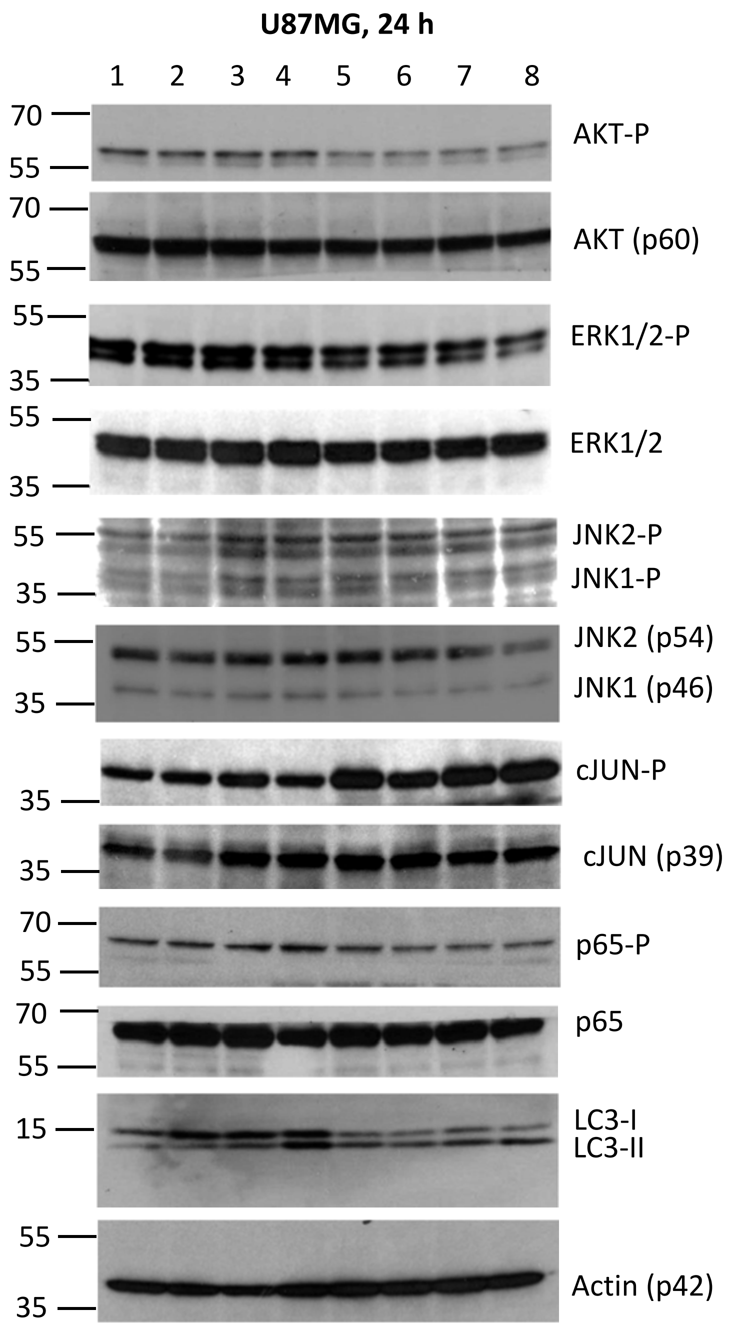

Figure 3a

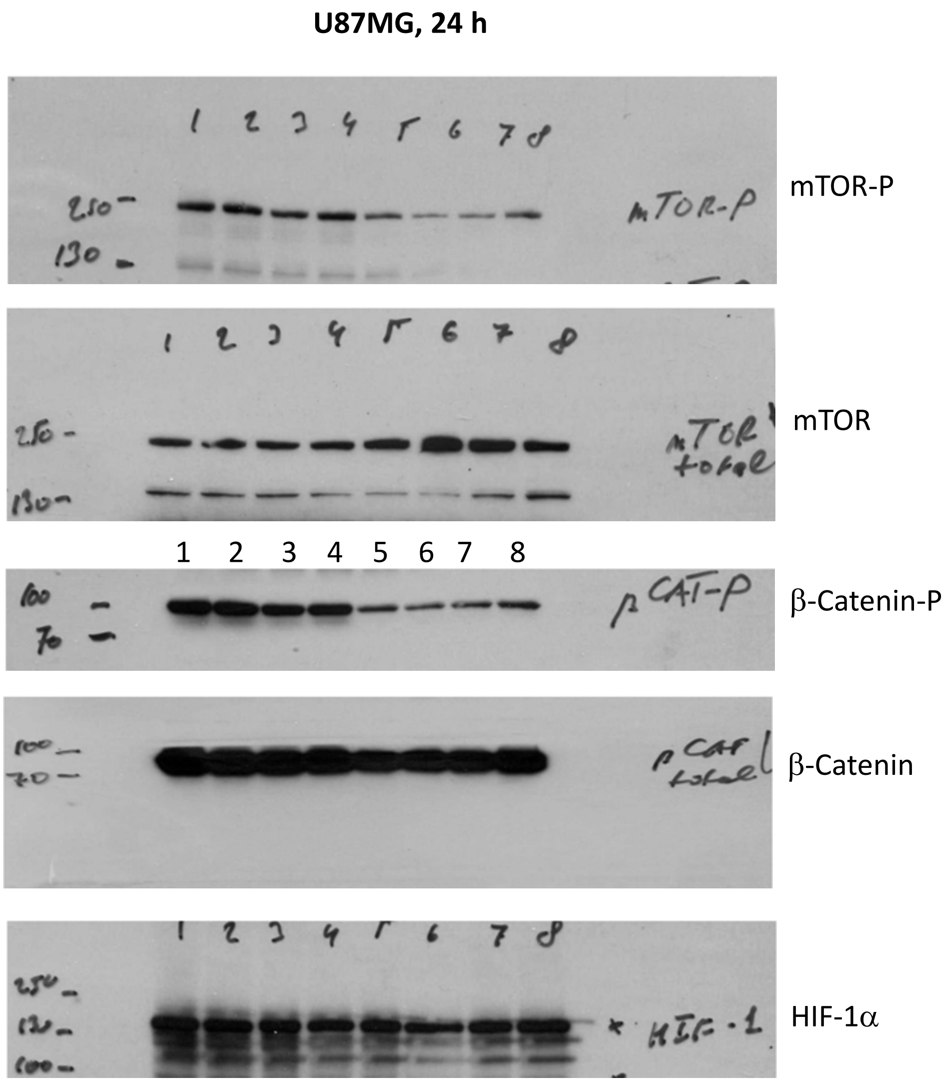

Figure 3a (addition)

Figure S3a. The originals of Western blots for Figure 3a.

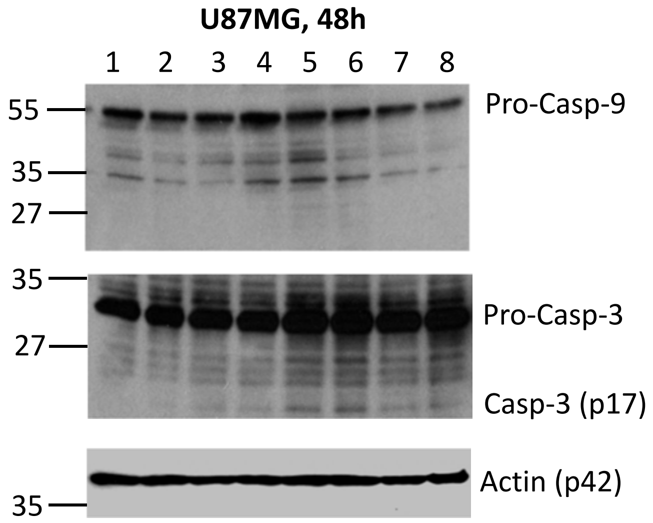

Figure 3b

Figure S3b. The originals of Western blots for Figure 3b.

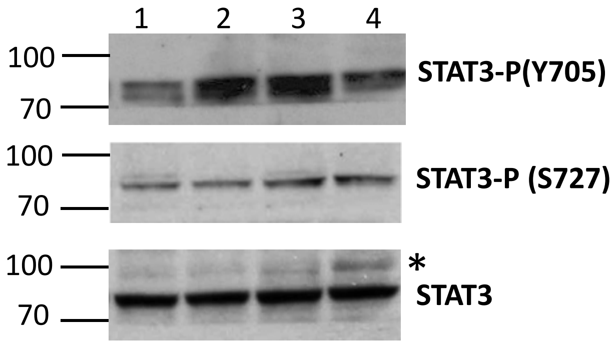

Figure 4f

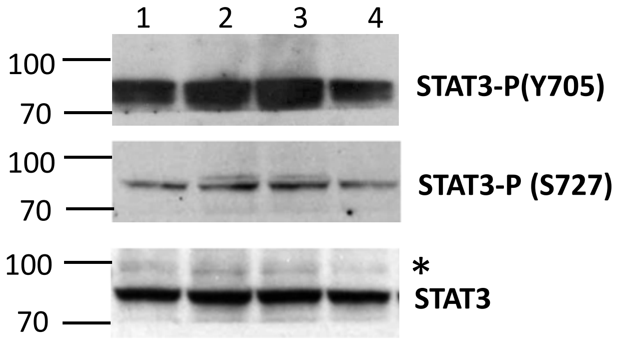

Figure 4g

Figure S4. The originals of Western blots for Figure 4f and 4g demonstrate total and phosphorylated STAT3 levels. Star indicates non-specific band.

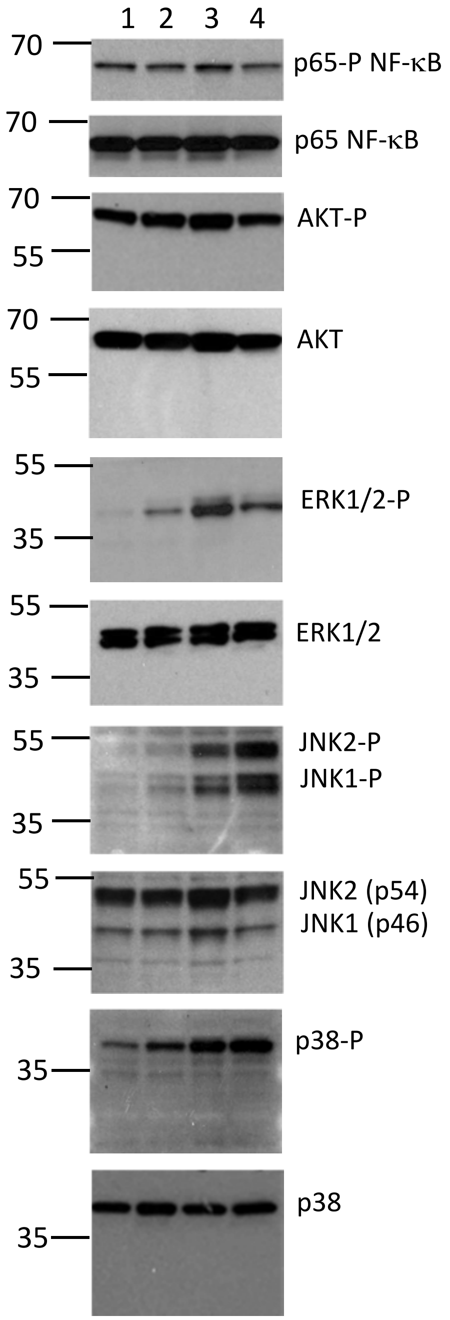

Figure 6a

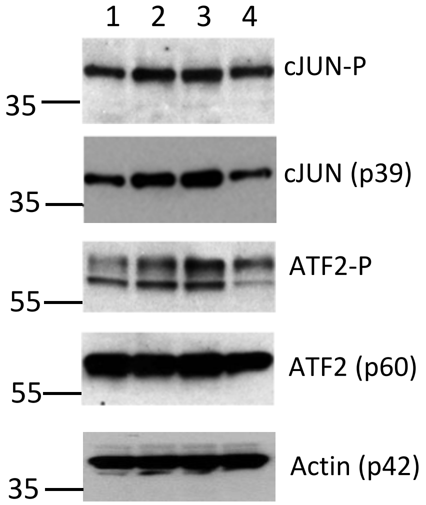

Figure 6a

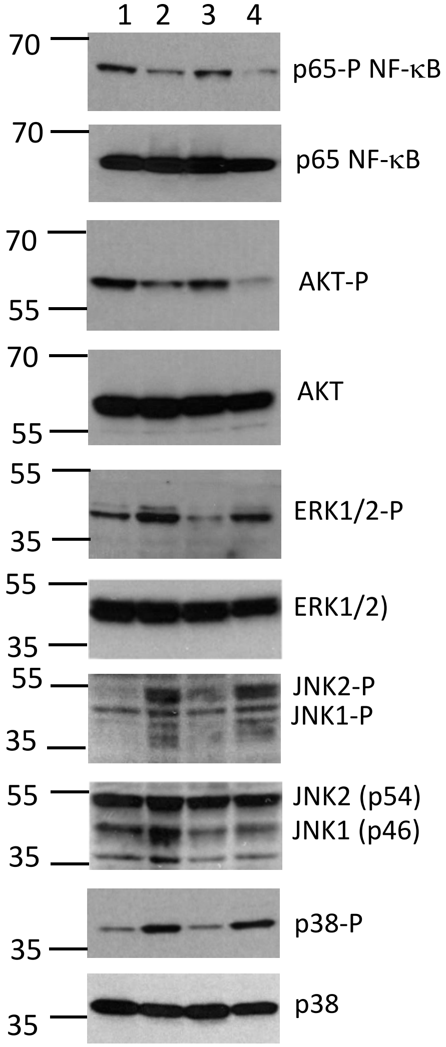

Figure 6b

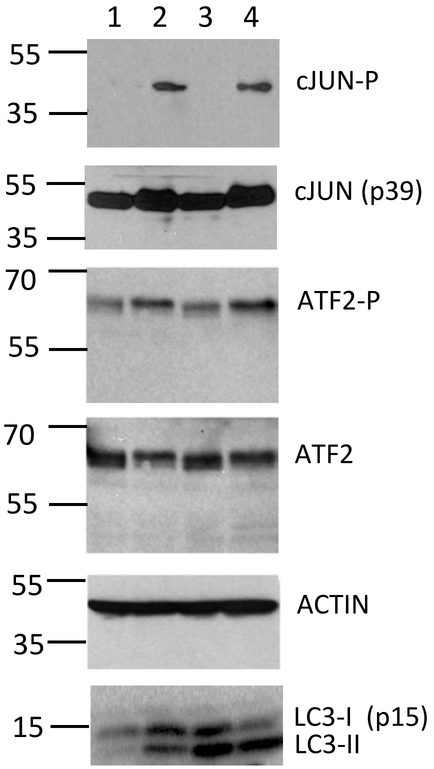

Figure 6b

Figure S6. The originals of Western blots for Figure 6. 24 h after treatment (Figure 6b), JNK1/2-P demonstrates increased levels of low molecular weight products, probably, as a result of degradation.

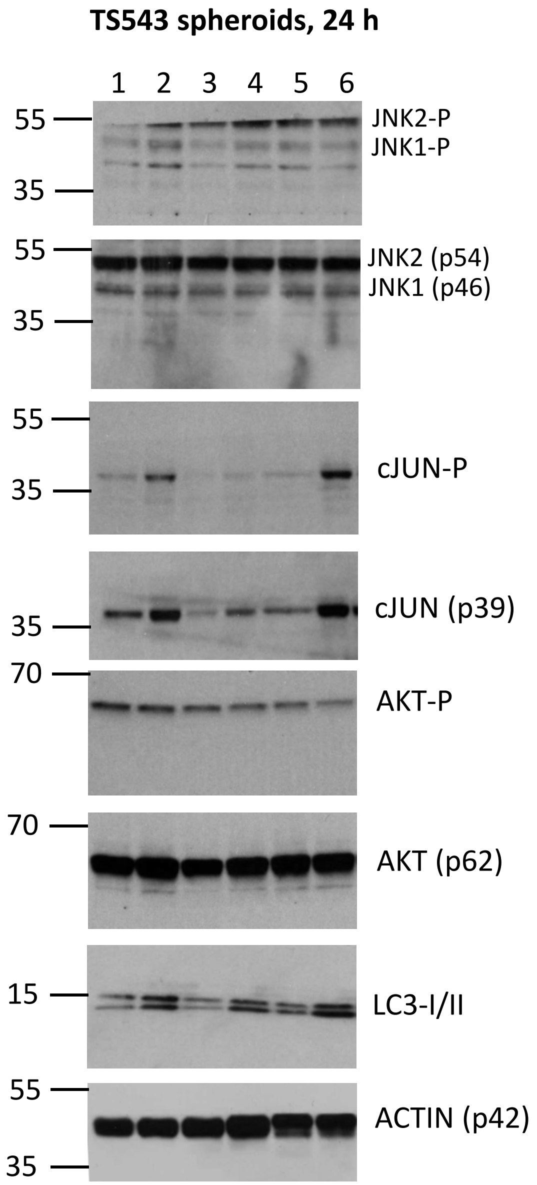

Figure S7. The originals of Western blots for Figure 7a.

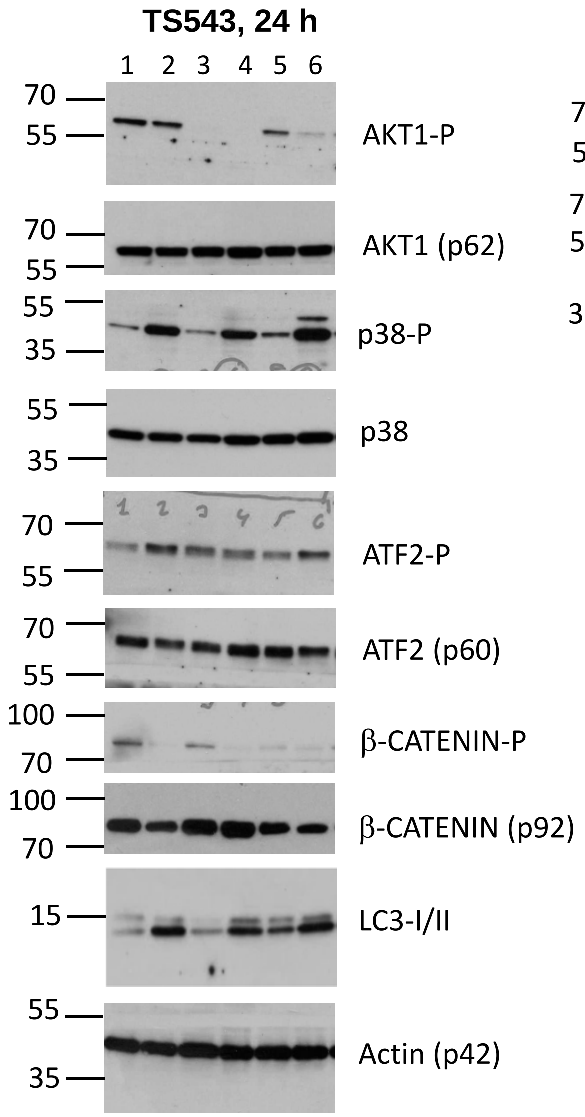

Figure 8a

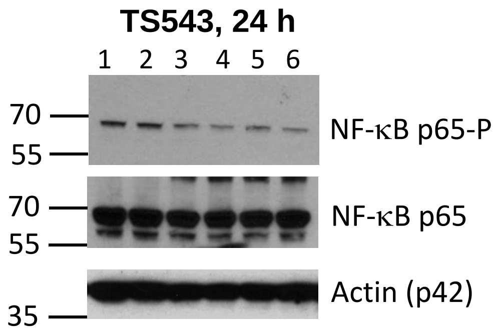

Figure 8b

Figure S8. The originals of Western blots for Figure 8a and 8b.

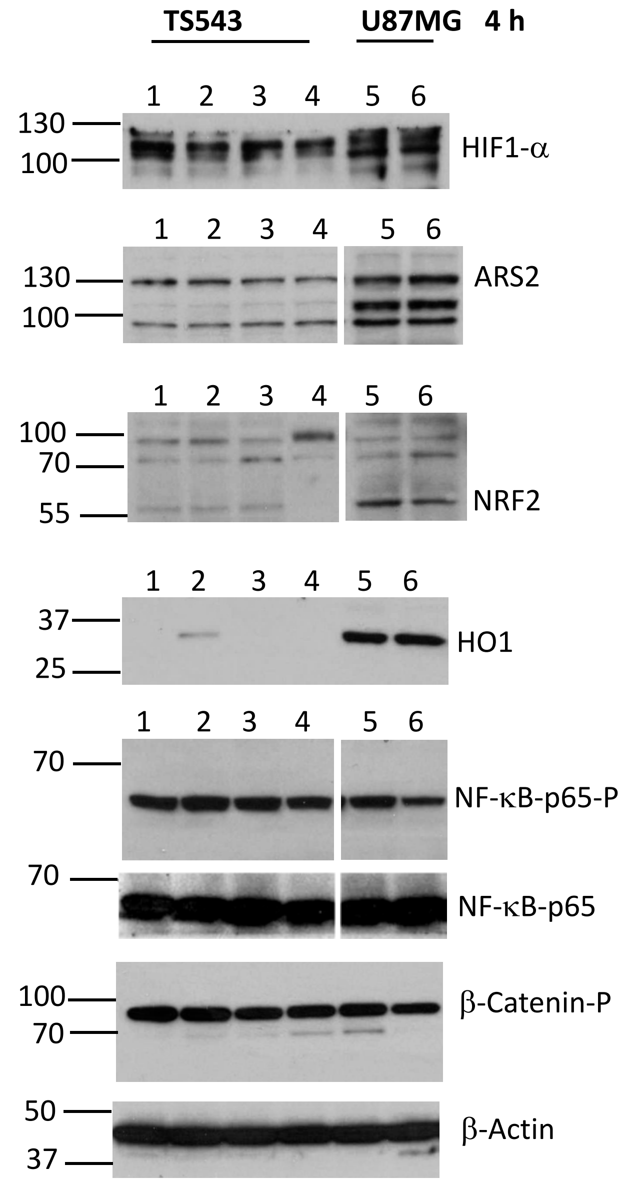

Figure S10. The originals of Western blots for Figure 10c. Two center lanes in gel blots for ARS2, NRF2, NF-κB-p65-P and NF-κB p65 (which contain proteins after additional treatments non-used in this paper) were removed.
